# Supplementary material for: Intention and Attitude to Accept a Pertussis Cocooning Vaccination among Chinese Children’s Guardians: A Cross-Sectional Survey
Source: Int J Environ Res Public Health. 2022 Dec 5;19(23):16282. doi: 10.3390/ijerph192316282 (PMC9740915; doi:10.3390/ijerph192316282)
Supplement: Supplementary file 1 [file ijerph-19-16282-s001.zip › ijerph-2055042-supplementary.pdf]

LEGENDS

Figure 1. A theoretical framework for accepting a pertussis cocooning vaccination originated from a literature review and Reasoned Action Approach.

Figure 2. The distribution of the sample provinces in the map of China, and the gray area represents the sample provinces.

Figure 3. Box plots of 5-point Likert-scales mean scores of attitude, subjective norm, and perceived control in the positive intention group and negative intention group. Data are median (central line), interquartile range (box margins), 5–95% percentile (whiskers), and outliers (dots).

Figure 4. Hierarchical logistic regression analysis of the determinants of intention to accept pertussis cocooning vaccination among guardians. Abbreviations: OR, odds ratio; CI, confidence interval.

Figure 5. Univariate and multivariate linear regression analysis of the determinants of guardians' attitude on pertussis cocooning vaccination. Note: CI, confidence interval. [a]Measured on a five-point Likert scale; low-high. [b] For each point increase on the Likert scale of the determinant, the intention changes with the value of the β. [c]one point is awarded for each correct answer. $R^2 = 35\%$.

Table 1. Characteristics of guardians by intention to accept a pertussis cocooning vaccination (N = 762). Note: [a]junior college or below, [b]Bachelor degree or above, [c]≤ 5000 CNY monthly household income, [d]> 5000 CNY monthly household income. It refers to net income.

Supplementary Table S1. An overview of the measured items within intention, psychosocial determinants of intention, and determinants of attitude toward pertussis cocooning vaccination, and responses in sample areas.

Items	Mean ± SD
**Intention**	
You would accept a pertussis cocooning vaccination in the first month after delivery if it was offered.	4.44 ± 0.927
**Psychosocial determinants of intention**	
**Attitude**	
As an adult, you are at risk of contracting pertussis.	3.65 ± 1.306
As a newborn, your child is, or was, at risk of contracting pertussis.	3.72 ± 1.367
It is safe to vaccinate adults against pertussis.	4.04 ± 1.057
Adult pertussis vaccination can effectively prevent pertussis.	4.09 ± 1.013
Total[a]	3.86 ± 0.863
**Subjective norm**	
How likely are you to receive the adult pertussis vaccine if recommended by your healthcare provider？	3.87 ± 1.090
If a friend or family member recommends adult pertussis vaccine to you, how likely are you to get it?	3.70 ± 1.146
If your child's teacher or principal recommends the adult pertussis vaccine, how likely are you to get it?	3.83 ± 1.093
If adult pertussis vaccine is recommended by the media (TV, Internet, magazines, leaflets), how likely are you to get this vaccine?	3.59 ± 1.183
Most parents like you get their adult pertussis vaccination as soon as possible after birth.	4.41 ± 1.081
Total[a]	3.82 ± 0.916
**Perceived control**	
If you were to be offered a pertussis cocooning vaccination in the first month after delivery, you trust you would be able to get vaccinated.	4.41 ± 0.929
If you were to be offered a pertussis cocooning vaccination, your current financial situation would allow you to get vaccinated.	3.97 ± 1.117
Total[a]	4.19 ± 0.859
**Determinants of attitude**	
**Risk perception**	

If you have not had an adult pertussis vaccination, how likely you think your child is to get pertussis?	3.17 ± 1.201
If your child has pertussis, how serious do you think it is?	4.41 ± 0.870
If you have not had an adult pertussis vaccination, the probability that you think you have contracted pertussis?	2.90 ± 1.180
If you have pertussis, how serious do you think it is?	4.02 ± 0.992
If you have pertussis, how likely you think you are to pass it on to your child?	4.00 ± 1.028
If you pass pertussis to your child, how would you feel?	4.66 ± 0.723
If you receive adult pertussis vaccination, you think you are likely to experience side effects.	2.67 ± 1.153
Are the side effects serious?	2.81 ± 1.147
Total[a]	3.58 ± 0.588
**General vaccination beliefs**	
Do you think you should get vaccinated?	4.78 ± 0.635
Infection (not death) is better than vaccination.	4.46 ± 1.155
Vaccines are offered merely for the sake of the medical industry.	4.34 ± 1.158
Following government policy for vaccination to be.	4.73 ± 0.709
Total[a]	3.17 ± 0.499
**Outcome expectation**	
If you accept a pertussis vaccine in the first month after delivery you protect my baby against pertussis.	4.42 ± 0.944
You think that implementing pertussis cocooning is too expensive comparing to the effects it will have.	3.16 ± 1.197
Total[a]	3.79 ± 0.803
**Moral norm**	
If a pertussis vaccine is offered, you will specifically get it to protect your child.	4.46 ± 0.905
Do you think it is your responsibility to get your child vaccinated against pertussis as soon as possible after birth?	4.48 ± 0.887
Total[a]	4.47 ± 0.844
**knowledge**	
For which age group is pertussis most dangerous?	0.40 ± 0.489
What is the most common source of infection for newborns？	0.57 ± 0.496
What is our country current pertussis vaccinal program?	0.34 ± 0.475
Have you heard of the pertussis cocooning vaccination?	0.42 ± 0.494
Do you think this immunization strategy is sufficient to protect your child from pertussis?	0.66 ± 0.474
Do you support the implementation of this immunization strategy in our country?	0.89 ± 0.312
Total	3.28 ± 1.370

Note: We used a five-point Likert scale to measure intention, psychosocial determinants of intention, and determinants of attitude (low-high); however, in the

knowledge dimension, one point was awarded for each correct answer. [a]Items for each participant were then averaged into a single measure, mean ± standard deviation.
